# TORC1 Inhibition by Rapamycin Promotes Antioxidant Defences in a *Drosophila* Model of Friedreich’s Ataxia

**DOI:** 10.1371/journal.pone.0132376

**Published:** 2015-07-09

**Authors:** Pablo Calap-Quintana, Sirena Soriano, José Vicente Llorens, Ismael Al-Ramahi, Juan Botas, María Dolores Moltó, María José Martínez-Sebastián

**Affiliations:** 1 Department of Genetics, University of Valencia, Burjassot, Valencia, Spain; 2 Department of Molecular and Human Genetics, Baylor College of Medicine, Houston, Texas, United States of America; 3 CIBERSAM, INCLIVA, Valencia, Spain; Sant Joan de Déu Children's Hospital, SPAIN

## Abstract

Friedreich’s ataxia (FRDA), the most common inherited ataxia in the Caucasian population, is a multisystemic disease caused by a significant decrease in the frataxin level. To identify genes capable of modifying the severity of the symptoms of frataxin depletion, we performed a candidate genetic screen in a *Drosophila* RNAi-based model of FRDA. We found that genetic reduction in TOR Complex 1 (TORC1) signalling improves the impaired motor performance phenotype of FRDA model flies. Pharmacologic inhibition of TORC1 signalling by rapamycin also restored this phenotype and increased the lifespan and ATP levels. Furthermore, rapamycin reduced the altered levels of malondialdehyde + 4-hydroxyalkenals and total glutathione of the model flies. The rapamycin-mediated protection against oxidative stress is due in part to an increase in the transcription of antioxidant genes mediated by *cap-n-collar* (*Drosophila* ortholog of *Nrf2*). Our results suggest that autophagy is indeed necessary for the protective effect of rapamycin in hyperoxia. Rapamycin increased the survival and aconitase activity of model flies subjected to high oxidative insult, and this improvement was abolished by the autophagy inhibitor 3-methyladenine. These results point to the TORC1 pathway as a new potential therapeutic target for FRDA and as a guide to finding new promising molecules for disease treatment.

## Introduction

Friedreich’s ataxia (FRDA), an autosomal recessive disease, is the most common inherited ataxia among Caucasians [1]. It is a multisystemic disease affecting the central and peripheral nervous systems and other non-neural organs, resulting in multiple signs and symptoms [2]. This incapacitating condition exhibits an irreversible progression that confines a patient to a wheelchair and leads to early death. FRDA is caused by a partial loss of *FXN* function [3], with the vast majority of patients carrying an intronic GAA expansion mutation in both alleles of this gene [4]. *FXN* codifies frataxin, a mitochondrial protein that is highly conserved through evolution [5] and whose deficiency results in several biochemical disturbances. Major alterations include impaired iron-sulphur cluster biogenesis, dysfunction of respiratory chain complexes and aconitase, mitochondrial iron accumulation and increased oxidative stress sensitivity [6].

A growing amount of data from patient samples and different model organisms of the disease suggest that oxidative stress plays an important role in the pathophysiology of FRDA. Biomarkers of oxidative damage, such as lipid peroxidation products, have been frequently found in patient samples and in a *Drosophila* model of the disease [7,8]. Increased levels of reactive oxygen species (ROS) have also been reported in FRDA lymphoblasts [9] and in mouse, *Drosophila* and yeast models [10–12]. In addition, frataxin depletion enhances the sensitivity to different pro-oxidant agents in FRDA cells, mice, *Drosophila melanogaster*, *Caenorhabditis elegans* and yeast [13–17]. Furthermore, a reduction in the ability to promote antioxidant defences has been reported in cultured fibroblasts from FRDA patients [18] and in the dorsal root ganglia from YG8R frataxin-deficient mice [19].

To date, there is no cure for FRDA, but several strategies for the discovery of effective therapeutics are being developed or tested in clinical trials (http://www.curefa.org/pipeline.html). These strategies seek to increase frataxin expression and to reduce the biochemical consequences of its deficiency, such as oxidative damage. Important progress has been achieved in frataxin replacement therapies [20,21], as well as in treatments directed to increasing protein levels [22]. Genetically manipulable organisms such as *Drosophila* are acquiring increased significance for medical and pharmaceutical research as valuable tools for testing potential therapies. The identification of the *Drosophila FXN* ortholog, *fh*, [23] led to the development of fly models of FRDA that can be used to explore frataxin function [24] and to provide *in vivo* evidence of a functional equivalence for human and fly frataxins [25]. These models have contributed to a comprehensive characterisation of the phenotype associated with frataxin deficiency [8,11,15,26–28]. Recently, we have validated the use of *Drosophila* as an experimental tool to screen for therapeutic molecules to treat FRDA and proposed that early treatments using the antioxidant idebenone and the iron chelator deferiprone may be advantageous to slow down the disease progression [29]. In addition the molecule methylene blue has been suggested for the treatment of the heart dysfunction in FRDA [30]. These findings stimulate further work using *Drosophila* to find new pharmacological drugs that may be relevant to this disease.

Here, we conducted a genetic screen of candidate genes related to FRDA pathophysiology to identify new therapeutic targets for this disease. We found that downregulation of TOR Complex 1 (TORC1) function suppresses the impaired motor performance of our *Drosophila* model of FRDA [15]. To evaluate the therapeutic efficacy of TORC1 inhibition, we used rapamycin, a lipophilic macrolide that acts as an inhibitor of the TOR kinase [31]. This treatment was able to increase the motor performance and survival of frataxin knockdown flies and could also induce an improvement in the oxidative status and an increase in the ATP levels.

## Materials and Methods

### *Drosophila melanogaster* strains

The *UAS*-*fh*RNAi line was previously generated in our laboratory and produces a reduction of up to 70% of frataxin mRNA when expressed ubiquitously using the *actin*-*Gal4* driver; this reduction is compatible with a normal development [15]. The *y* ^*1*^ w*, *w* ^1118^, *UAS*-*GFP*, *actin-Gal4*, *nos-Gal4*; *UAS*-*GFP*-*LC3*, *UAS*-*foxo*-*GFP*, *cnc*-*EGFP*, *UAS-S6k* ^*STDETE*^ (here referred as *S6k* ^*CA*^) and *Thor* ^*2*^ (here referred as *4E-BP* ^*LOF*^) strains were obtained from the Bloomington Stock Center. *y* ^*1*^ w*; *actin-Gal4* and *w* ^1118^; *actin-Gal4* flies were used as controls, while *UAS-fh*RNAi; *actin-Gal4* flies were used as FRDA model flies (here referred as *fh*RNAi).

### Culture conditions and drug treatments

*Drosophila* stocks were maintained at 25°C under a 16/8 hour light/dark cycle on standard cornmeal agar medium. The media named “RAP”, “3-MA” and “RAP + 3-MA” were prepared with, respectively rapamycin at 1 μM (LC Laboratories), 3-methyladenine at 67 μM (Sigma-Aldrich), and both rapamycin at 1 μM and 3-MA at 67 μM. All compounds were previously dissolved in dimethylsulfoxide (DMSO; Sigma-Aldrich) at a final concentration of 0.1% (v/v). The medium named “DMSO” only contained this compound at 0.1% (v/v) and was used as control medium. Crosses were conducted at 25°C in the supplemented media. F_1_ flies of the appropriate genotype were transferred to fresh vials containing the compound every 3 days.

### Genetic screen

The *UAS*-*fh*RNAi; *actin*-*Gal4* flies (*fh*RNAi flies) were crossed at 28°C with approximately 300 lines, including RNAi lines from the Vienna Drosophila Resource Center and loss-of-function and overexpression lines for candidate genes from the Bloomington Stock Center. We focused on candidate pathways implicated in FRDA pathophysiology comprising metal homeostasis, response to oxidative stress, apoptosis and autophagy. Motor performance tests were conducted as described previously [32] for the identification of genetic modifiers of frataxin depletion. We recorded the number of flies that climbed to a height of 11.5 cm.

### Climbing and survival assays

Groups of fifteen 7-day-old males were transferred into vials of 1.5 cm in diameter and 25 cm in height. The height reached from the bottom of the vial by each fly in a period of 10 s was recorded with a camera. For each genotype, approximately 100 flies were tested. The results are expressed in percentage, taking as 100% the mean climbing speed of control flies in the DMSO medium. Lifespan was measured starting with 100 adult males of each genotype and by recording the number of living flies every 3 days. Survival under hyperoxia was measured using 30 adult males exposed to a constant flux of 99.5% oxygen under a low positive pressure from day 1 to day 4 after eclosion from the puparium. Three replicates were performed, and the results showed the percentage of dead flies after 4 days of hyperoxia.

### GFP-LC3 quantification in larvae

The *UAS-fh*RNAi and *y* ^*1*^ w* lines were crossed with the *nos* - *Gal4*; *UAS*-*GFP*-*LC3* strain, which expresses the microtubule-associated protein 1A/1B-light chain 3 (LC3) as a fusion protein with GFP under the control of the *nanos* promoter (a marker for autophagy [33]). Fat bodies of third instar larvae were imaged with a fluorescence microscope (Leica DM 2500; Leica Microsystems) using a x40 objective. Larvae were maintained in normoxia or subjected to one day of hyperoxia before the dissections. The number of fluorescent dots per field was counted automatically using the tools of ImageJ software (National Institutes of Health, USA). The results are expressed in percentage, taking as 100% the average number of dots for control flies in the DMSO medium.

### Biochemical assays

Biochemical assays were conducted in triplicate or quadruplicate with thirty 7-day-old males of the appropriate genotype. ATP levels were determined using the ATP Detection Reagent of the Mitochondrial ToxGlo Assay (Promega). Flies were homogenised in a buffer of 0.25 M Sucrose; 10 mM HEPES-NaOH pH 7.4; 0.1% Triton X-100 (v/v), Na_3_VO_4_ 5 mM, and the extract was centrifuged at 1000 g for 10 min at 4°C. The luminescence of the supernatant was measured using a Tecan Infinite M200 PRO luminometer (Tecan Group). ATP levels were normalised to the total protein, which was measured using the BCA assay. The results are expressed in percentage, taking as 100% the ATP level of control flies in the DMSO medium.

The concentration of malondialdehyde (MDA) + 4-hydroxyalkenals (HAE) was measured using the Bioxytech LPO-586 Kit (Oxis International). Flies were homogenised in a buffer of 50 mM Tris-HCl at pH 7.4 with 5 mM butylated hydroxytoluene, and the extract was centrifuged at 3000 g for 10 min at 4°C. A_586_ measurements were performed in a Spectronic Genesys 5 spectrophotometer (Milton Roy). MDA + HAE levels were normalised to the protein amount determined by the Bradford assay. The results are expressed in nmol of MDA + HAE per μg of protein.

The total concentration of GSH, including both the reduced and oxidised forms, was measured using a Bioxytech GSH-420 Spectrophotometric Assay Kit (Oxis International). Flies were homogenised in trichloroacetic acid, and the extract was centrifuged at 3000 g for 10 min at 4°C. A_420_ was measured in a Spectronic Genesys 5 spectrophotometer (Milton Roy). The obtained data were normalised to the total protein determined by the Bradford assay, and the results are expressed in mmol of total GSH per μg of protein.

For measurements of the aconitase activity in hyperoxia, flies were incubated in normoxia for 5 days and then treated with 99.5% oxygen for 2 days. Aconitase activity was determined from the whole-fly extracts using the Bioxytech Aconitase-340 Spectrophotometric Assay Kit (Oxis International) as previously described [29]. A_340_ was measured using a Tecan Infinite M200 PRO luminometer (Tecan Group), and the results are expressed in percentage, taking as 100% the aconitase activity of control flies in DMSO medium.

### Quantitative real-time PCR (RT-qPCR)

Total RNA was extracted from 7-day-old males using a miRNeasy Mini Kit (Qiagen). RNA was converted into cDNA with Expand Reverse Transcriptase (Roche) and oligo-dT primers. Amplification was conducted using the Step One Plus Real-Time PCR System (Applied Biosystems) and Power SYBR Green (Applied Biosystems). The following primers were used for the transcript amplification of the different genes: *frataxin homolog (fh)*, 5′ACACCCTGGACGCACTGT3′ and 5′CCAGGTTCACGGTTAGCAC3′; *Adenylyl cyclase 76E* (*Ac76E*), 5’CGATCAAATAGCTCAGGAGAACCA3’ and 5’ CATTTATGCCGGTCGCCTCA3’; *cap-n-collar* (*cnc*), 5'CACGTTTTCAAGCTCACCAC3' and 5'TCCCTGCAGCACACACAAT3'; *Catalase (Cat*), 5'GTTCGAGTGTTTCTAAATTCTGGTT3' and 5'GTGGTAATGGCACCAGGAGAA3'; *forkhead box sub-group O (foxo*), 5'CCCACCGGCAAAATCAACAA3' and 5'CCTCGCCAGCCCAAAAGATA3'; *Glutamate-cysteine ligase catalytic subunit (Gclc*), 5'GAGAGCGAAACAGAGTGACGA3' and 5'GAACTGATTGACGCCATGCT3'; *Glutathione S transferase D1* (*GstD1*), 5'TACATCGCGAGTTTCACAACAG3' and 5'CAGGTTGAGCAGCTTCTTGTT3'; *Peroxiredoxin 3* (*Prx3*), 5'CCGATTTCAAGGGTCTGGCT3' and 5'CAACAATTTCGGTGGGGCAA3'; *Sestrin* (*Sesn*), 5'CCCCAGTTCCACGATCACTT3' and 5'CGCTTCACCAGATACGGACA3'; *Superoxide dismutase* (*Sod*), 5' GAACAGGAGAGCAGCGGTA3' and 5'TACGGATTGAAGTGCGGTCC3'; *Superoxide dismutase 2* (*Sod2)*, 5'CAGATATGTTCGTGGCCCGT3' and 5'CGGCAGATGATAGGCTCCAG3'. The *Ribosomal protein L32* gene (*RpL32*) was used as an internal control and was amplified using the 5′CCAAGCACTTCATCCGCCACC3′ and 5′GCGGGTGCGCTTGTTCGATCC3′ primers. The results were analysed using the Step One Plus software v2.0 (Applied Biosystems). The gene expression levels are relative to the internal control, and the relative quantification of each cDNA was calculated in quadruplicate experiments using the Ct method. The results are expressed as the fold change of relative gene expression compared with that for control flies in the DMSO medium.

### Nuclear isolation and GFP quantification

*fh*RNAi flies were crossed with the *UAS*-*foxo*-*GFP* and *cnc*-*EGFP* lines. For *UAS*-*foxo*-*GFP*, third instar larvae were collected as the co-expression with *fh*RNAi resulted in adult semi-lethality; whereas 7-day-old males were analysed for *cnc*-*EGFP*. Flies with the genotypes *actin*-*Gal4; UAS*-*foxo*-*GFP* and *actin*-*Gal4; cnc*-*EGFP*, respectively, were used as controls. Total extractions and nuclear fractions were isolated following the procedure described previously [34], and GFP fluorescence was measured using a Tecan Infinite M200 PRO luminometer (Tecan Group). The results are expressed as the nuclear/total fluorescence ratio, considering the ratio of the control flies in the DMSO medium as 1.

### Statistical Analysis

Statistical analyses were performed using GraphPad Prism 5.03 software (GraphPad software). Kaplan-Meier survival plots were analysed using semiparametric log rank tests. For the comparison of means, we performed an unpaired nonparametric Student's *t* test. In all cases, values of *P*<0.05 were considered statistically significant. Error bars represent standard error of the mean (SEM).

## Results

### TORC1 pathway genetically interacts with frataxin

**Fig 1:**
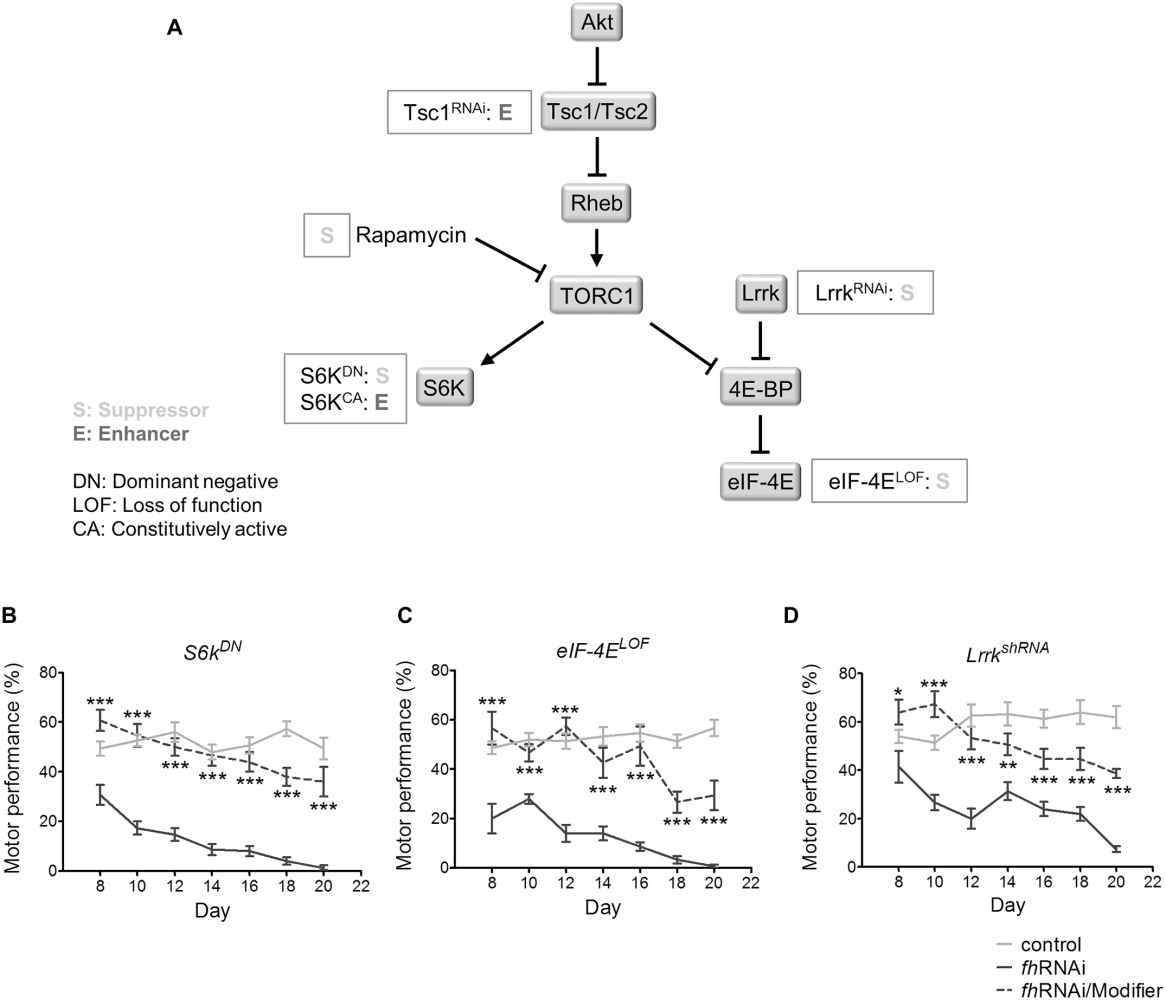
Genetic reduction of TORC1 signalling improves the motor performance of frataxin knockdown flies. (A) Gene modifiers of frataxin knockdown identified in *Drosophila* and their position within the TORC1 signalling pathway. (B-D) Improvement of motor performance of *fh*RNAi flies by the effect of a dominant negative allele of *S6k* (B), a loss of function allele of *eIF*-*4E* (C) and a shRNA against *Lrrk* (D). Motor performance is expressed as the percentage of flies that climbed to a height of 11.5 cm. Control (*w^1118^*; *actin*-*Gal4* flies), *fh*RNAi (*UAS*-*fh*RNAi; *actin*-*Gal4* flies), *fh*RNAi/Modifier (*fh*RNAi flies carrying the corresponding allele of the modifier). Asterisks represent the statistical significance between the *fh*RNAi and *fh*RNAi/Modifier flies for every day. *P<0.05, **P<0.01, ***P<0.001. Error bars represent SEM.

To identify genetic modifiers that might modulate the phenotypes caused by frataxin knockdown in *Drosophila*, we conducted a genetic screen of candidate pathways implicated in FRDA pathophysiology. Specifically, we examined the effect of knockdown, loss and gain-of-function alleles corresponding to metal homeostasis, response to oxidative stress, apoptosis and autophagy pathways. We then conducted motor performance tests to determine whether these alleles suppress the motor impairment of the *fh*RNAi flies. The screen revealed four modifiers from the TORC1 signalling pathway: the tuberous sclerosis complex protein 1 (*Tsc1*), the protein kinase S6K (*S6k*), the eukaryotic translation initiation factor 4E (*eIF-4E*) and the Leucine-rich repeat kinase (*Lrrk*) (Fig 1A).

One of the most important regulators of TORC1 activity is the tuberous sclerosis complex (TSC), which is a heterodimer that comprises the proteins TSC1 and TSC2. TSC1/2 negatively regulates TORC1 signalling by inactivating the Ras homolog enriched in brain ortholog (*Rheb*) GTPase. The simultaneous knockdown of *Tsc1* and frataxin resulted in semi-lethality, whereas the expression of the RNAi for *Tsc1* with the *actin*-*Gal4* driver had no effect on viability in control flies. *S6k* and *eIF-4E* are downstream targets of TORC1. Expression of a dominant-negative form of S6K [35] improved the motor performance of the *fh*RNAi flies (Fig 1B). In contrast, the expression of a constitutively active version of S6K [35] produced a detrimental effect when combined with frataxin knockdown by inducing semi-lethality. Regarding *eIF-4E*, a loss of function mutation suppressed the impaired motor performance phenotype of the *fh*RNAi flies (Fig 1C). In all cases, the *S6k* and *eIF-4E* alleles on their own had no effect on the viability or motor performance of control flies. We also demonstrated that knocking down the *Lrrk* suppresses the frataxin knockdown phenotype (Fig 1D). *Lrrk* is the *Drosophila* ortholog of the human *LRRK2*, and dominant pathogenic mutations in this gene cause the most common familial forms and some sporadic cases of Parkinson’s disease [36]. In *vitro* studies show that the eIF-4E binding protein (4E-BP) is a substrate of *Lrrk* [37], (Fig 1A). Genotypes of the *Drosophila* strains corresponding to these genetic interactors are shown in S1 Table.

### Rapamycin improves the motor performance and lifespan deficits of the *fh*RNAi flies

**Fig 2:**
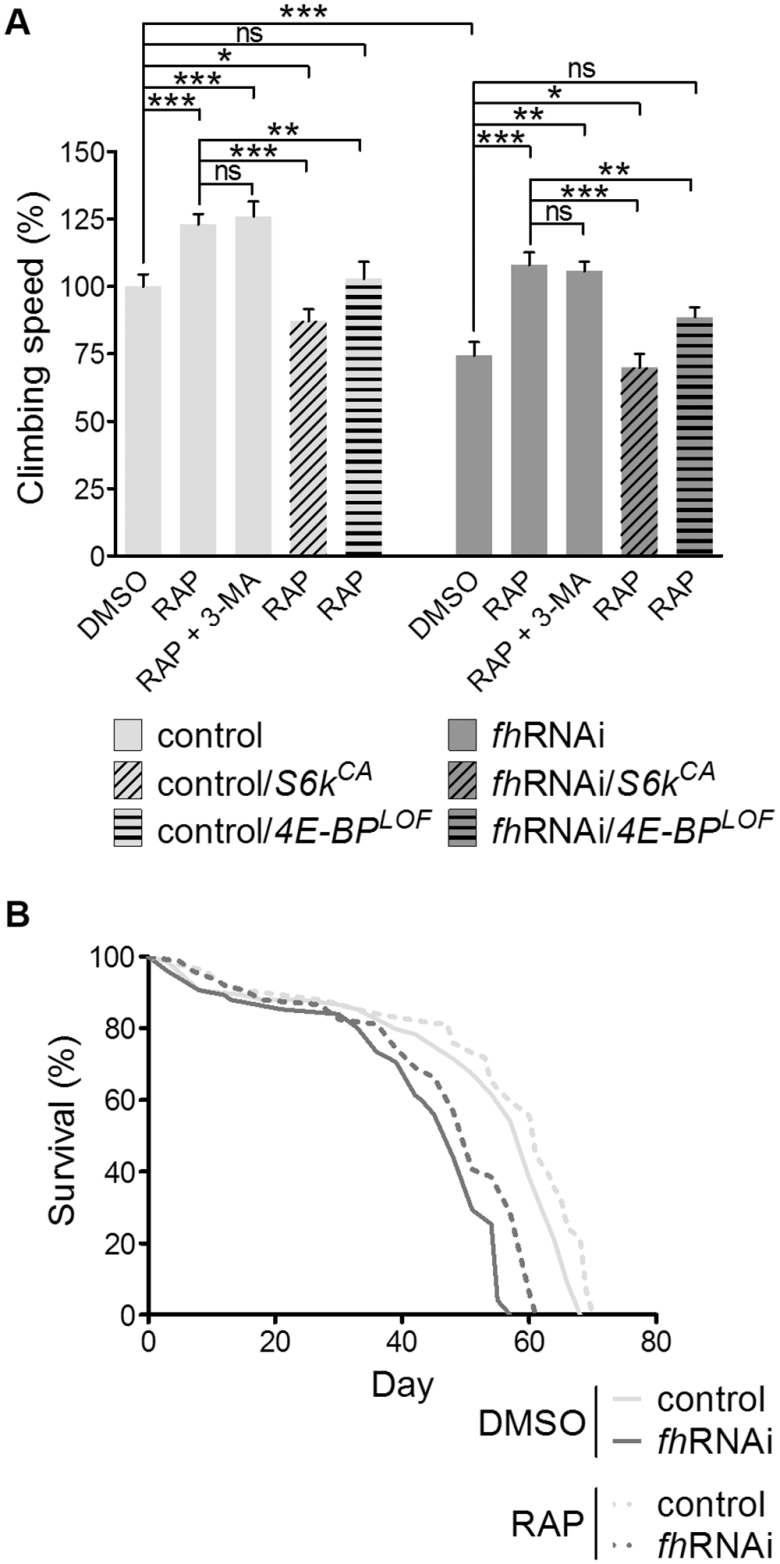
Rapamycin increases climbing speed and survival. (A) Rapamycin treatment increases the climbing speed of 7-day-old males in both the control and FRDA fly groups, and this effect is not affected by the addition of the autophagy inhibitor 3-MA. A constitutively active allele of S6K (*S6k^CA^*) and a loss of function allele of 4E-BP (*4E-BP^LOF^*) prevent rapamycin from increasing the climbing speed. Climbing speed is expressed in percentage (mean climbing speed of control flies in the DMSO medium is 100%). ns: non-significant, *P<0.05, **P<0.01, ***P<0.001. Error bars represent SEM. (B) Survival is also increased in control (*P* = 0.0116) and FRDA flies (*P* = 0.0004) after the drug treatment. Control (y^1^w*; actin-Gal4 flies), control/*S6k^CA^* (control flies carrying the *S6k^CA^* allele), control/*4E-BP^LOF^* (control flies carrying the *4E-BP^LOF^* allele), *fh*RNAi (*UAS-fh*RNAi; *actin-Gal4* flies), *fh*RNAi /*S6k^CA^* (model flies carrying the *S6k^CA^* allele), *fh*RNAi /*4E-BP^LOF^* (model flies carrying the *4E-BP^LOF^* allele).

Because the reduction of TORC1 activity decreases S6K and eIF-4E activities [38], we tested whether pharmacologic inhibition of this complex with rapamycin would also improve the phenotypes of our *Drosophila* FRDA model. First, we tested the effect of 1 μM rapamycin on the motor performance of the frataxin knockdown flies. This concentration had been previously proved effective in *Drosophila* [39] and did not provoke the negative effect on viability that we found for the higher concentrations. Here, we used the climbing speed of flies as it provides a more accurate assessment of the motor performance. In DMSO medium, 7-day-old *fh*RNAi flies showed a 25% decrease in climbing speed compared with controls raised in the same medium. Rapamycin induced the recovery of the motor performance phenotype of the frataxin knockdown flies up to control levels (Fig 2A). Because another feature of our *Drosophila* model of FRDA is a shortened lifespan [15], we also tested the effect of rapamycin on this phenotype. It has been reported that rapamycin increases the lifespan in *Drosophila* and other organisms [40]. Accordingly, 1 μM rapamycin produced a slight but statistically significant increase in the lifespan of both control (*P* = 0.0116) and *fh*RNAi (*P* = 0.0004) flies (Fig 2B).

To confirm the inhibitory effect of rapamycin on TORC1 activity, we measured the developmental time needed by flies to reach the adult stage. It has been shown that, in conjunction with the insulin/IGF signalling pathway, TORC1 controls the larval development in *Drosophila*, matching the speed of growth to the nutrient availability. A reduction in the amount of food reduces TORC1 signalling in the fat body and the prothoracic gland and increases the time needed by the individuals to reach the pupae stage [41]. We observed that the rapamycin treatment increased, by approximately one day, the mean time needed by both control and *fh*RNAi individuals to reach the adult stage (S1 Fig). This result indicated that rapamycin reduces TORC1 signalling similarly to the food restriction effect and that the rapamycin concentration used could efficiently modify the TORC1 activity.

Finally, to test whether the suppression by rapamycin of the motor performance and lifespan phenotypes of *fh*RNAi flies was an artefact caused by interference with the GAL4/UAS system, we verified that rapamycin had no effect on the expression of a GFP reporter (S2 Fig). RT-qPCR of the transcript for frataxin showed that rapamycin did not alter the level of the *fh* mRNA either in the control or in the frataxin knockdown flies (S3 Fig).

### Rapamycin protects against oxidative stress in the FRDA model

Oxidative stress plays a central role in the pathophysiology of FRDA, as shown in patients and in cellular and animal models of the disease [7–12], including our *Drosophila* model. In this context, the impairment of motor performance and survival exhibited by the *fh*RNAi flies were ameliorated after treatment using the antioxidant idebenone [29]. *fh*RNAi flies also show an increased sensitivity to external oxidative stress, as indicated by an enhanced reduction in motor performance and lifespan [15]. Taking into account these data, we asked whether rapamycin might be suppressing the FRDA toxicity in part by decreasing oxidative stress.

**Fig 3:**
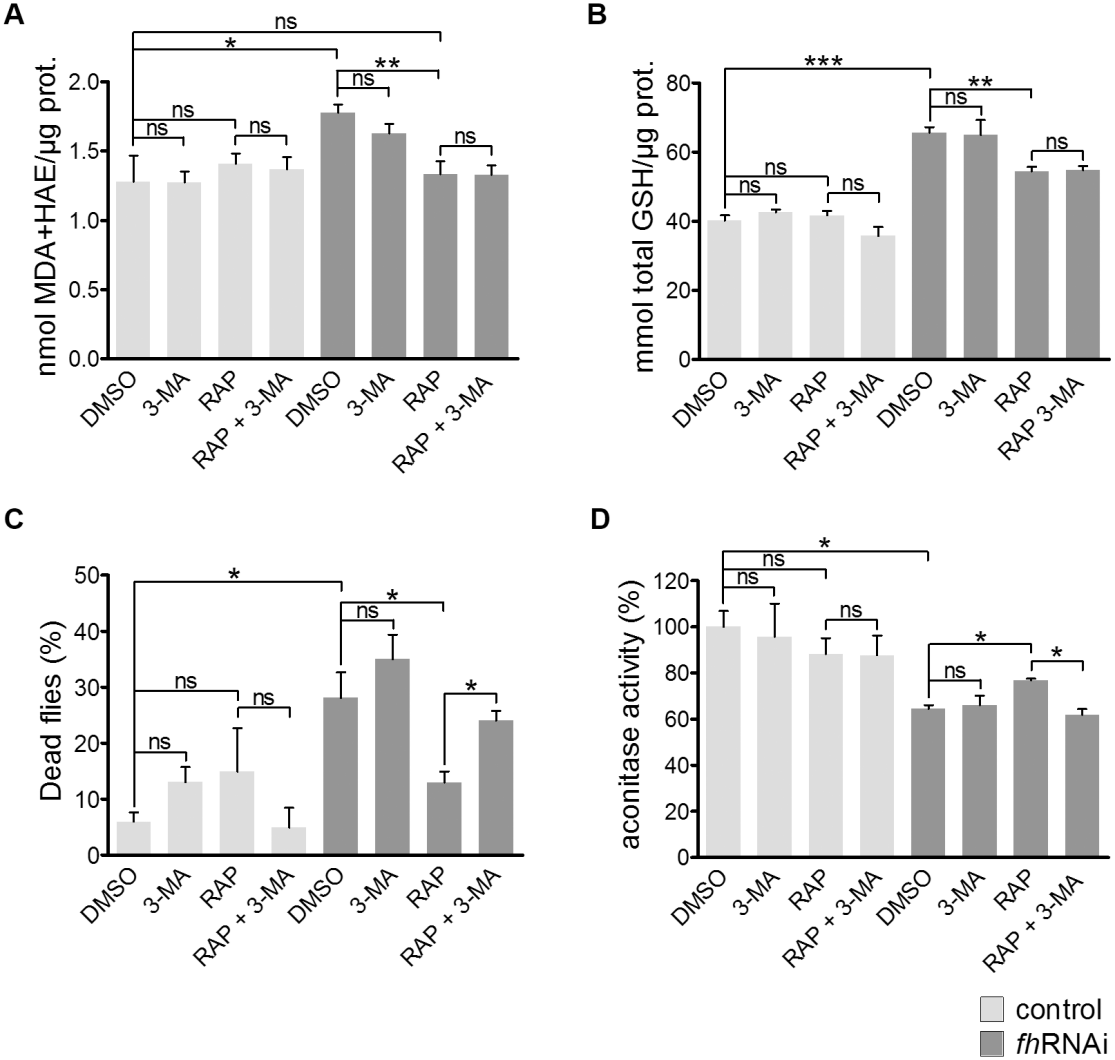
Rapamycin increases oxidative stress protection. In the normoxic condition, rapamycin reduces the altered levels of malondialdehyde + 4-hydroxyalkenals (A) and total glutathione (B) of model flies without requiring autophagy induction as the addition of 3-MA has no effect. In hyperoxia, rapamycin improves the survival of FRDA flies (C) and increases the aconitase activity (D). The addition of 3-MA abolishes these effects in model flies, highlighting the relevance of autophagy in this highly oxidative condition. Control (*y* ^*1*^ w*; *actin-Gal4* flies), *fh*RNAi (*UAS-fh*RNAi; *actin-Gal4* flies). ns: non-significant, *P<0.05, **P<0.01, ***P<0.001. Error bars represent SEM.

We monitored the effect of rapamycin on the levels of malondialdehyde (MDA) + 4-hydroxyalkenals (HAE) and total glutathione, two markers of oxidative stress. As expected, *fh*RNAi flies show a higher amount of MDA + HAE compared with that of control flies in the DMSO medium (Fig 3A). Interestingly, rapamycin restored the MDA + HAE levels in the *fh*RNAi flies, whereas rapamycin had no effect on the controls. As shown in Fig 3B, *fh*RNAi flies had higher levels of total glutathione than did control flies and rapamycin produced a significant reduction in the total amount of glutathione in the *fh*RNAi flies but did not affect the total glutathione levels in the controls. Therefore, the inhibition of TORC1 with rapamycin seems to ameliorate the oxidative stress injury in frataxin knockdown flies, resulting in a decrease in the altered MDA + HAE and total glutathione levels.

### Autophagy induction by rapamycin is not critical except in hyperoxic conditions

It is well established that rapamycin treatment leads to the activation of autophagy through the inhibition of TORC1 [31]. Thus, we asked whether autophagy might play a role in the protection against oxidative stress conferred to the *fh*RNAi flies by rapamycin. For this purpose, we used the 3-MA compound, which inhibits Vps34 (vacuolar protein sorting 34), a class III phosphoinositide 3-kinase that is essential for autophagosome biogenesis [42]. First, we confirmed that at the concentrations used in this study, rapamycin and 3-MA were effective as inducers and inhibitors of autophagy, respectively. As shown in S4 Fig, rapamycin induces the formation of autophagosomes, which were labelled with GFP-LC3, in control and frataxin knockdown flies, and the addition of 3-MA decreased the number of GFP-LC3 dots.

Next, we tested the effect of autophagy inhibition on the levels of MDA + HAE and total glutathione. No changes were detected between the RAP and the RAP + 3-MA media (Fig 3A and 3B), indicating an autophagy-independent effect for rapamycin. In addition, the beneficial effect of rapamycin on the motor performance was also autophagy-independent (Fig 2A). These data show that even though autophagy is induced by rapamycin, autophagy has no important protective effect in these conditions.

Then, we asked whether autophagy induction by rapamycin is beneficial for flies under external oxidative stress. We assessed the effect of rapamycin on the survival of *fh*RNAi flies incubated in a hyperoxic atmosphere for 4 days. In the DMSO medium, we observed higher mortality in *fh*RNAi flies (28%) than in controls (6%). Rapamycin reduced the number of dead *fh*RNAi flies but had no significant effect on the survival of the controls (Fig 3C). Interestingly, the decreased lethality observed in hyperoxia conditions was abolished by the addition of 3-MA (Fig 3C), suggesting that autophagy is indeed necessary for the protective effect of rapamycin on *fh*RNAi flies in hyperoxia. In this condition, rapamycin and 3-MA were also effective as an inducer and inhibitor of autophagy, respectively (S4 Fig).

To further confirm this hypothesis, we measured the activity of aconitase during hyperoxia. Under this experimental condition, the reduction of enzyme activity was significantly higher in the *fh*RNAi flies than in the control flies [15]. We observed that the aconitase activity increased in the rapamycin-treated *fh*RNAi flies and that this increase was also abolished by the addition of 3-MA (Fig 3D). Together, these data suggest that the autophagy induction by rapamycin is required for the protection against highly oxidative conditions; however, other mechanisms downstream of TORC1 should act in conditions of endogenous production of ROS in the FRDA model.

### Rapamycin enhances antioxidant defences increasing the nuclear translocation of Cnc

**Fig 4:**
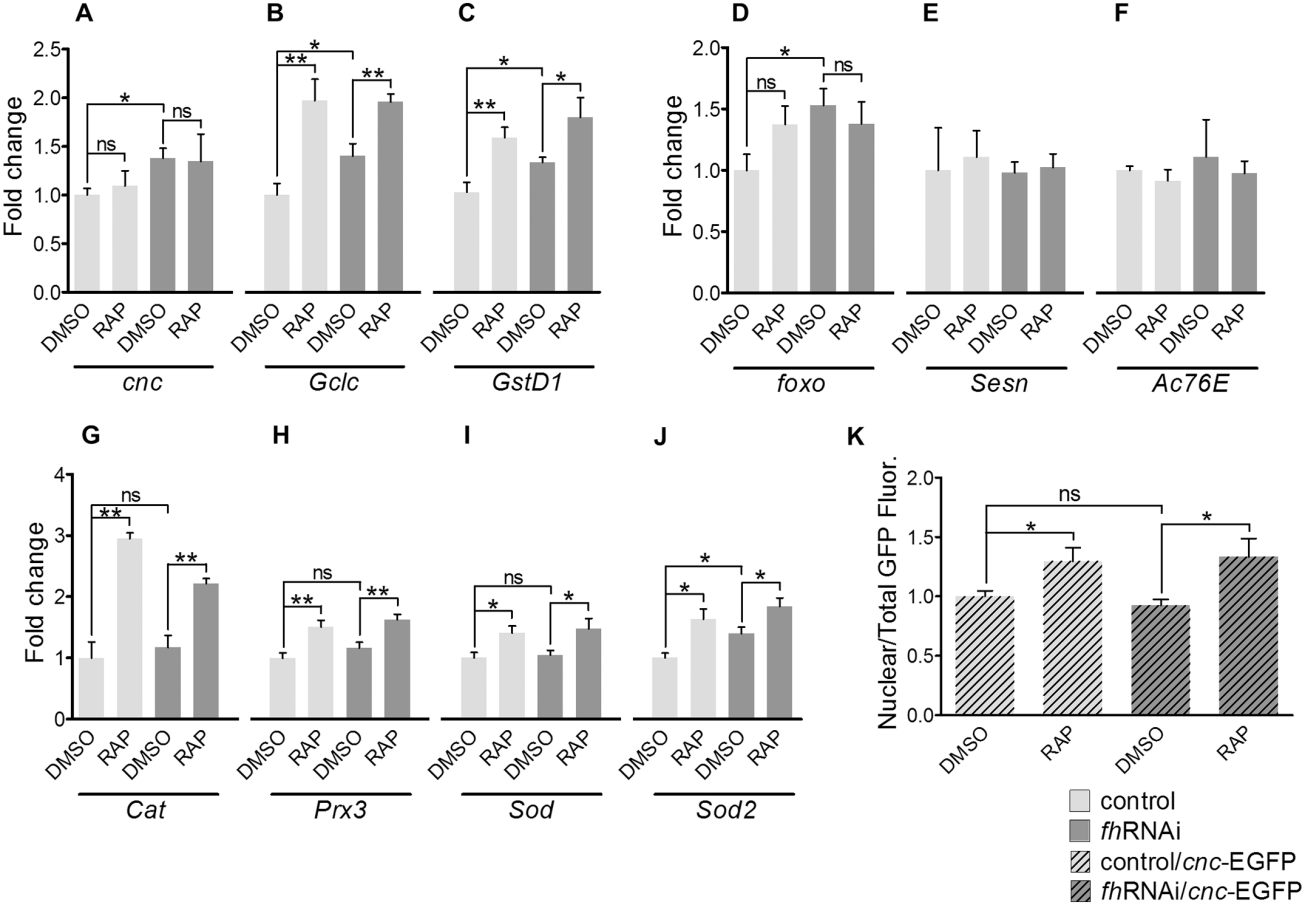
Rapamycin increases the expression of antioxidant genes under the control of Cnc. (A) *cnc* expression is not affected by rapamycin, but the transcription of two Cnc targets, *Gclc* (B) and *GstD1*(C), is higher in flies treated with the compound. (D-F) Rapamycin does not affect neither *foxo* expression nor the expression of the two FOXO targets *Sesn* and *Ac76E*. (G-J) Rapamycin increases the mRNA level of *Cat*, *Prx3*, *Sod* and *Sod2*, which are subjected to overlapping regulation from FOXO and Cnc transcription factors. (K) Rapamycin also increases the fraction of Cnc located in the nucleus. Control (*y* ^*1*^ w*; *actin-Gal4* flies), *fh*RNAi (*UAS-fh*RNAi; *actin-Gal4* flies), control/*cnc*-EGFP (control flies expressing a *cnc* allele tagged with the EGFP) and *fh*RNAi/*cnc*-EGFP: (*fh*RNAi flies expressing a *cnc* allele tagged with the EGFP). ns: non-significant, *P<0.05, **P<0.01. Error bars represent SEM.

TORC1 modulates the function of several transcription factors that, in turn, control the transcription of important antioxidant genes [43,44]. To determine whether rapamycin transcriptionally induces endogenous antioxidant defences, we analysed the expression of two key transcription factors implicated in antioxidant protection (*foxo* and *cnc*) and four well-known target genes (*Sesn* and *Ac76E* for FOXO; *Gclc* and *GstD1* for Cnc). In the DMSO medium, we observed higher levels of the *foxo* (53% increase) and *cnc* (38% increase) transcripts in the *fh*RNAi flies than in the controls. Rapamycin did not modify the expression of these genes at the transcriptional level (Fig 4A and 4D). The FOXO target genes *Sesn* and *Ac76E* showed no differences between the control and *fh*RNAi flies in both DMSO and RAP medium (Fig 4E and 4F). In contrast, the transcript levels of the Cnc target genes were higher in the *fh*RNAi flies than in the controls when both were cultured in the DMSO medium (40% for *Gclc* and 34% for *GstD1*). Rapamycin increased the expression of these genes in both the control and *fh*RNAi flies (Fig 4B and 4C). Rapamycin also increased the mRNA level of *Cat*, *Prx3*, *Sod* and *Sod2* (Fig 4G–4J), which encode important enzymes that protect the cell from oxidative damage and are subjected to overlapping regulation of FOXO and Cnc transcription factors.

To explain the augmented expression of the Cnc target genes by rapamycin, we searched for cellular localisation of Cnc using an EGFP-tagged *cnc* allele. We found a higher nuclear/cellular fluorescence ratio of Cnc-EGFP after rapamycin treatment in both the control and *fh*RNAi flies (Fig 4K). No differences were observed in the case of a FOXO-GFP fused protein (S5 Fig). These results indicate that rapamycin enhances the protection against oxidative stress by inducting endogenous antioxidant defences and that this effect is mediated, at least in part, by an increase in the nuclear translocation of the transcription factor Cnc.

### Rapamycin increases the availability of ATP through 4E-BP

**Fig 5:**
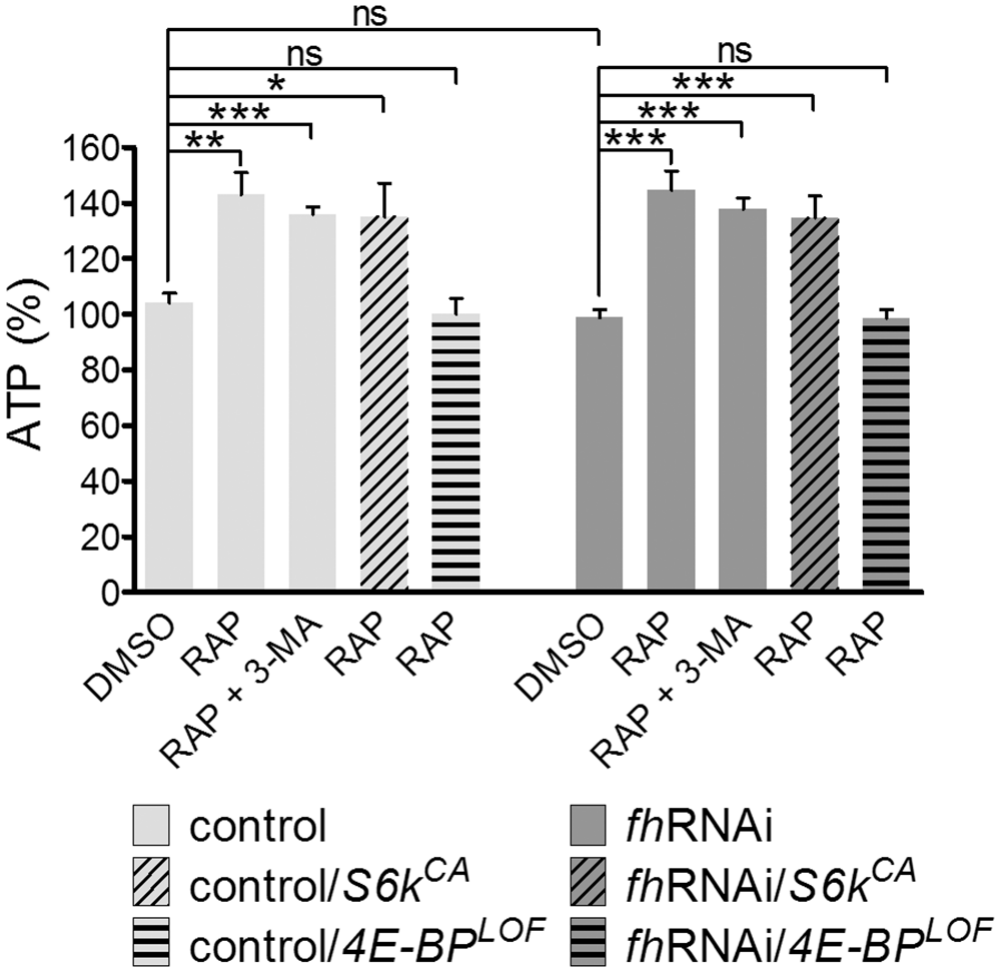
Rapamycin increases ATP levels both in control and *fh*RNAi flies. Rapamycin is able to increase ATP levels in control and *fh*RNAi flies even with the addition of the autophagy inhibitor 3-MA or in flies carrying a constitutively active allele of S6K (*S6k* ^*CA*^). In flies carrying a 4E-BP loss of function allele (*4E-BP* ^*LOF*^), rapamycin cannot increase ATP levels. Control (*y* ^*1*^ w*; *actin-Gal4* flies), *fh*RNAi (*UAS-fh*RNAi; *actin-Gal4* flies), control/*S6k* ^*CA*^ (control flies carrying the *S6k* ^*CA*^ allele), control/ *4E-BP* ^*LOF*^ (control flies carrying the *4E-BP* ^*LOF*^ allele), *fh*RNAi */S6k* ^*CA*^ (model flies carrying the *S6k* ^*CA*^ allele), *fh*RNAi/*4E-BP* ^*LOF*^ (model flies carrying the *4E-BP* ^*LOF*^ allele). ns: non-significant, *P<0.05, **P<0.01, ***P<0.001. Error bars represent SEM.

A pathological reduction of frataxin levels results in an impairment of ATP synthesis [45,46]. We measured the ATP levels in whole-fly extracts, and we did not find significant differences when comparing *fh*RNA*i* and control flies in the DMSO medium (Fig 5). Because different *Drosophila* tissues have distinct sensitivity to frataxin depletion [15], it is possible that the reduced ATP levels may be restricted to these tissues. Interestingly, rapamycin treatment increased the ATP levels (Fig 5) in both the control (41% increase) and frataxin knockdown flies (37% increase). This increase may contribute to the beneficial effect of rapamycin on the FRDA phenotype. To identify the pathway downstream of TORC1 involved in the ATP increase, we combined the rapamycin treatment with 3-MA, a constitutively active allele of S6K or a loss of function allele of 4E-BP, and we measured the ATP levels in the three cases. We observed that the 4E-BP mutation prevented rapamycin from increasing the ATP levels, while the expression of the constitutively active S6K and the inhibition of autophagy had no effect on that increase. These results indicate that rapamycin ameliorates the ATP availability in flies through the 4E-BP and that inactivation of S6K or autophagy induction after TORC1 inhibition is not critical in this process [47,48]. Nevertheless, the modulation of the activity of both S6K and 4E-BP by TORC1 but not autophagy is indeed required for the rapamycin-mediated recovery of motor performance of *fh*RNAi flies (Fig 2A).

## Discussion

TOR is an evolutionarily conserved protein that senses and integrates various environmental and intracellular signals to regulate growth and homeostasis in all eukaryotic cells. It functions by forming two structurally and functionally different multiprotein complexes, TORC1 and TOR Complex 2 (TORC2). TORC1 is better described and regulates many major cellular functions, including protein synthesis, lipid biogenesis and autophagy. TORC1 is sensitive to inhibition by rapamycin treatment [49].

In this study, we identified several components of the TORC1 pathway as modifiers of frataxin knockdown phenotypes in *Drosophila melanogaster*. We found that a genetic reduction in TORC1 signalling activity suppresses the impaired motor performance phenotype of the *fh*RNAi flies. Accordingly, genetic activation of TORC1 signalling produced a detrimental effect when combined with frataxin knockdown by inducing semi-lethality. Thus, we tested the therapeutic usefulness of a pharmacologic inhibition of TORC1 in the *fh*RNAi flies, using the natural macrolide rapamycin [31]. We observed that rapamycin restored the motor performance of frataxin knockdown flies to normal levels, in agreement with the genetic reduction of *S6k* and *eIF-4E*. Rapamycin also increased the mean and maximum survival of the *fh*RNAi flies similarly as it did in controls. It is well known that TORC1 inhibition prolongs the lifespan of different species [40,50] and such effect is extended to *fh*RNAi flies.

The expression of dominant-negative forms of TOR and S6K and TOR inhibition by rapamycin provides flies with resistance to oxidative stress, whereas increased Rheb-TOR-S6K signalling sensitises flies to this type of stress and promotes early senescence of locomotor activity [50, 51]. Furthermore, it has also been reported in *Drosophila* that 4E-BP is important for survival under different types of stress, including oxidative insult [52]. In particular, high levels of oxidative stress markers have been found in FRDA patient samples as well as in several models of the disease [9,53–55]. In agreement with these results, we found an increased amount of MDA and HAE in the *fh*RNAi flies; these lipid peroxidation compounds are produced after the breakdown of unstable polyunsaturated fatty acid peroxides. The *fh*RNAi flies also showed increased levels of total GSH, a molecule with antioxidant function that reduces hydrogen and lipid peroxides when it is oxidised to its disulphide form, GSSG. Interestingly, rapamycin reduced significantly the MDA + HAE and total glutathione levels in the *fh*RNAi flies, restoring to some extent the normal situation observed in control flies. Recently, a similar result has been reported in the yeast frataxin knockout model in which rapamycin reduces ROS production [56].

The inhibition of TOR signalling by rapamycin has been shown to be protective against toxicity in several cell and animal models of neurodegenerative diseases. In some cases, this protective effect of rapamycin appears to be autophagy-dependent, particularly in neurodegenerative diseases associated with aggregation-prone mutant proteins [57]. However, our results indicated that in a normoxic condition, autophagy is not the main mechanism by which rapamycin protects *fh*RNAi flies against the ROS injury caused by frataxin deficiency. Our data show that neither the rescue of motor performance nor the protection against ROS induced by rapamycin were affected by the chemical inhibitor of autophagy 3-MA in normoxia.

TORC1 inhibition by rapamycin increases the transcript levels of genes involved in the free radical scavenging and Nrf2-mediated oxidative stress response in mouse adult stem cells [43] and in *C*. *elegans* [44]. We studied the possible effect of rapamycin on the activity of the transcription factors FOXO and Cnc (the *Drosophila* ortholog of the mammalian Nrf2), which control the expression of many genes involved in resistance against different types of stresses [44,58]. Our results showed that rapamycin increased the transcription of antioxidant genes dependent upon Cnc but not FOXO, and that this effect is mediated by an increase in the nuclear translocation of Cnc. Therefore, it may be the origin of the protective effect of rapamycin against oxidative stress caused by frataxin reduction in the *fh*RNAi flies. There are still many aspects of Nrf2 regulation that remain poorly understood. Nevertheless, several regulation mechanisms have been already described, both dependent and independent of the Keap1 protein (recently reviewed in [59]). Some of these mechanisms could explain the increase in Cnc activity mediated by rapamycin. Protein kinase C is able to disrupt the association between Nrf2 and Keap1, promoting the translocation of Nrf2 into the nucleus. GSK3β promotes Nrf2 ubiquitination and the degradation of the protein by the proteasome; thus, GSK3β inhibition can also contribute to Nrf2 activity. Both Protein kinase C activation and GSK3β inhibition can be triggered by an increase in PI3K-Akt signalling, and rapamycin is able to produce this effect by means of a negative feedback loop in TORC1 regulation [60,61] (S6 Fig). However, the actual mechanism by which rapamycin increases Cnc activity in FRDA model flies needs further research.

Interestingly, we found that autophagy becomes an important protective mechanism in *fh*RNAi flies subjected to a strong external oxidative insult. *fh*RNAi flies cultured in a hyperoxic environment show an enhanced reduction of motor performance and lifespan [15]. In this work, we observed that rapamycin improves the survival and aconitase activity of *fh*RNAi flies subjected to hyperoxia and that the beneficial effect of rapamycin decreases when 3-MA is added. Our results agree with other studies [39,50,62] in which autophagy is induced by rapamycin or by overexpressing/interfering *Atg* genes or components of the TORC1 signalling cascade to protect flies against strong external oxidative stress. Altogether, these data suggest that the autophagy induction by rapamycin operates as a cellular mechanism to protect against strong oxidative insults. However, in the oxidative conditions resulting from the endogenous ROS production in the *fh*RNAi flies, the protective effects of rapamycin are more likely to reside in its antioxidant properties rather than autophagy induction.

In conditions of frataxin depletion, several deficiencies in the mitochondrial electron transport chain result in impaired generation of ATP [45,46]. Reducing TORC1 activity may be beneficial for the energy status of frataxin-depleted cells because this signalling pathway activates specific regulatory mechanisms that can increase mitochondrial efficiency. Bonawitz et al. [47] reported that *tor*1 null yeast exhibit a higher rate of mitochondrial translation and steady-state abundance of several mitochondria-encoded OXPHOS components. In *Drosophila*, dietary restriction, whose effects are mediated to a great extent by TORC1, is capable of increasing the translation of genes involved in oxidative phosphorylation to ensure continued ATP generation, and this effect has been attributed to the TORC1 target 4E-BP [48]. In support of this idea, we found that inhibition of TORC1 by rapamycin increases the total ATP levels of both control and *fh*RNAi flies, which may contribute to the recovery of the motor performance and the slight increase in lifespan of the *fh*RNAi flies. We also found that 4E-BP is the key mediator in the increase of ATP levels after TORC1 inhibition by rapamycin. Finally, although much progress has been made in the understanding of TORC1 function, we cannot exclude the possibility that other unknown molecular mechanisms regulated by this critical signalling complex may be contributing to the recovery of the motor dysfunction of the rapamycin-treated *fh*RNAi flies.

Rapamycin is a well-described drug approved for human uses. This drug and its analogues (rapalogs) have important clinical applications in oncology and transplantation medicine. Ongoing clinical trials using rapalogs to treat different malignancies are providing an extensive body of data about the safety, tolerability and side effects of rapalogs [63]. In FRDA, lower doses of rapamycin may be beneficial combined with other drugs as antioxidants and iron chelators. It may enhance the advantages of either compound acting alone because none of the tested antioxidants or chelators has been proven to be sufficiently effective on the neurological symptoms of FRDA [64].

Our results show that the reduction of TORC1 signalling activity in the Drosophila model of FRDA rescues several phenotypes (impairment of motor abilities and reduced lifespan) that mimic the clinical features of this disease. These results point to the TORC1 pathway as a new potential therapeutic target for FRDA and as a guide to finding new promising molecules for disease treatment.

## Supporting Information

S1 Fig TORC1 inhibition by rapamycin increases development time to adulthood in control and *fh*RNAi flies. The time needed by individuals to eclose from the puparium was measured. The day the crosses were made was established as day zero. Parental flies were maintained in these vials for 2 days and then were removed. The results indicate the average time, in days, needed by individuals of F_1_ to complete the preadult development. Control (y^1^w*; *actin-Gal4* flies), *fh*RNAi (*UAS-fh*RNAi; *actin-Gal4* flies). ns: non-significant, ***P<0.001.
(TIF)

S2 Fig Rapamycin does not interfere with the GAL4/UAS system. Fluorescence from thirty 7-day-old males expressing GFP in a ubiquitous pattern (*UAS*-*GFP*; *actin*-*Gal4*) was measured as previously described in [29].
(TIF)

S3 Fig Rapamycin does not alter the level of *fh* mRNA neither in control nor in *fh*RNAi flies. Control (*y* ^*1*^ w*; *actin-Gal4* flies), *fh*RNAi(*UAS-fh*RNAi; *actin-Gal4* flies). ns: non-significant.
(TIF)

S4 Fig Effect of rapamycin and 3-MA on the induction and inhibition, respectively, of autophagosome formation. ns: non-significant, *P<0.05, **P<0.01, ***P<0.001. control/GFP-LC3 (UAS-GFP-LC3/+; Nos-Gal4/+) and *fh*RNAi/GFP-LC3: (UAS-GFP-LC3/UAS-*fh*RNAi; Nos-Gal4/+)
(TIF)

S5 Fig Rapamycin does not alter the fraction of FOXO-GFP located in the nucleus. control/*foxo*-GFP (control flies expressing a *foxo* allele tagged with the GFP) and *fh*RNAi/*foxo*-EGFP: (*fh*RNAi flies expressing a *foxo* allele tagged with the GFP).
(TIF)

S6 Fig Hypothesis of Nrf2 activation by rapamycin. Rapamycin might increase Nrf2 activity by mechanisms depending on PKC and GSK3β, triggered by a TORC1 negative feedback loop which may increase PI3K-Akt signalling.
(TIF)

S1 Table Genotypes of the *Drosophila* strains corresponding to the genetic interactors in the TORC1 pathway. (TIF)

## References

[1] Palau F, Espinós C. Autosomal recessive cerebellar ataxias. Orphanet J Rare Dis. 2006; 1: 47–65. 17112370 10.1186/1750-1172-1-47 PMC1664553

[2] Delatycki MB, Corben LA. Clinical features of Friedreich ataxia. J Child Neurol. 2012; 27: 1133–1137. 10.1177/0883073812448230 22752493 PMC3674491

[3] Campuzano V, Montermini L, Moltò MD, Pianese L, Cossée M, Cavalcanti F, et al Friedreich’s ataxia: autosomal recessive disease caused by an intronic GAA triplet repeat expansion. Science. 1996; 271: 1423–1427. 8596916 10.1126/science.271.5254.1423

[4] Monrós E, Moltó MD, Martínez F, Cañizares J, Blanca J, Vílchez JJ, et al Phenotype correlation and intergenerational dynamics of the Friedreich ataxia GAA trinucleotide repeat. Am J Hum Genet. 1997; 61: 101–110. 9245990 10.1086/513887 PMC1715858

[5] Gibson TJ, Koonin EV, Musco G, Pastore A, Bork P. Friedreich's ataxia protein: phylogenetic evidence for mitochondrial dysfunction. Trends Neurosci. 1996; 19: 465–468. 8931268 10.1016/S0166-2236(96)20054-2

[6] Schmucker S, Puccio H. Understanding the molecular mechanisms of Friedreich’s ataxia to develop therapeutic approaches. Hum Mol Genet. 2010; 19: R103–110. 10.1093/hmg/ddq165 20413654

[7] Emond M, Lepage G, Vanasse M, Pandolfo M. Increased levels of plasma malondialdehyde in Friedreich ataxia. Neurology. 2000; 55: 1752–1753. 11113241 10.1212/wnl.55.11.1752

[8] Navarro JA, Ohmann E, Sanchez D, Botella JA, Liebisch G, Moltó MD, et al Altered lipid metabolism in a Drosophila model of Friedreich's ataxia. Hum Mol Genet. 2010; 19: 2828–2840. 10.1093/hmg/ddq183 20460268 PMC7108586

[9] Napoli E, Taroni F, Cortopassi GA. Frataxin, iron-sulfur clusters, heme, ROS, and aging. Antioxid Redox Signal. 2006; 8: 506–516. 16677095 10.1089/ars.2006.8.506 PMC1805116

[10] Ristow M, Mulder H, Pomplun D, Schulz TJ, Muller-Schmehl K, Krause A, et al Frataxin deficiency in pancreatic islets causes diabetes due to loss of beta cell mass. J Clin Invest. 2003; 112: 527–534. 12925693 10.1172/JCI18107 PMC171391

[11] Anderson PR, Kirby K, Orr WC, Hilliker AJ, Phillips JP. Hydrogen peroxide scavenging rescues frataxin deficiency in a Drosophila model of Friedreich’s ataxia. Proc Natl Acad Sci. U.S.A. 2008; 105: 611–616. 10.1073/pnas.0709691105 18184803 PMC2206584

[12] Irazusta V, Cabiscol E, Reverter-Branchat G, Ros J, Tamarit J. Manganese is the link between frataxin and iron-sulfur deficiency in the yeast model of Friedreich ataxia. J Biol Chem. 2006; 281: 12227–12232. 16510442 10.1074/jbc.M511649200

[13] Wong A, Yang J, Cavadini P, Gellera C, Lonnerdal B, Taroni F, et al The Friedreich's ataxia mutation confers cellular sensitivity to oxidant stress which is rescued by chelators of iron and calcium and inhibitors of apoptosis. Hum Mol Genet 1999; 8: 425–30. 9949201 10.1093/hmg/8.3.425

[14] Al-Mahdawi S, Pinto RM, Varshney D, Lawrence L, Lowrie MB, Hughes S, et al GAA repeat expansion mutation mouse models of Friedreich ataxia exhibit oxidative stress leading to progressive neuronal and cardiac pathology. Genomics 2006; 88: 580–590. 16919418 10.1016/j.ygeno.2006.06.015 PMC2842930

[15] Llorens JV, Navarro JA, Martínez-Sebastián MJ, Baylies MK, Schneuwly S, Botella JA, et al Causative role of oxidative stress in a Drosophila model of Friedreich ataxia. FASEB J. 2007; 21: 333–344. 17167074 10.1096/fj.05-5709com

[16] Vázquez-Manrique RP, González-Cabo P, Ros S, Aziz H, Baylis HA, Palau F. Reduction of Caenorhabditis elegans frataxin increases sensitivity to oxidative stress, reduces lifespan, and causes lethality in a mitochondrial complex II mutant. FASEB J. 2006; 20: 172–174. 16293572 10.1096/fj.05-4212fje

[17] Lefevre S, Sliwa D, Auchre F, Brossas C, Ruckenstuhl C, Boggetto N, et al The yeast metacaspase is implicated in oxidative stress response in frataxin-deficient cells. FEBS Lett. 2012; 586: 143–148. 10.1016/j.febslet.2011.12.002 22155640

[18] Paupe V, Dassa EP, Goncalves S, Auchère F, Lönn M, Holmgren A, et al Impaired nuclear Nrf2 translocation undermines the oxidative stress response in Friedreich ataxia. PLoS One. 2009; 4: e4253 10.1371/journal.pone.0004253 19158945 PMC2617762

[19] Shan Y, Schoenfeld RA, Hayashi G, Napoli E, Akiyama T, Iodi Carstens M, et al Frataxin deficiency leads to defects in expression of antioxidants and Nrf2 expression in dorsal root ganglia of the Friedreich's ataxia YG8R mouse model. Antioxid Redox Signal. 2013; 19: 1481–1493. 10.1089/ars.2012.4537 23350650 PMC3797453

[20] Gimenez-Cassina A, Wade-Martins R, Gomez-Sebastian S, Corona JC, Lim F, Diaz-Nido J. Infectious delivery and long-term persistence of transgene expression in the brain by a 135-kb iBAC-FXN genomic DNA expression vector. Gene Ther. 2011; 18: 1015–1019. 10.1038/gt.2011.45 21490681

[21] Perdomini M, Belbellaa B, Monassier L, Reutenauer L, Messaddeq N, Cartier N, et al Prevention and reversal of severe mitochondrial cardiomyopathy by gene therapy in a mouse model of Friedreich's ataxia. Nat Med. 2014; 20: 542–547. 10.1038/nm.3510 24705334

[22] Soragni E, Xu C, Plasterer HL, Jacques V, Rusche JR, Gottesfeld JM. Rationale for the development of 2-aminobenzamide histone deacetylase inhibitors as therapeutics for Friedreich ataxia. J Child Neurol. 2012; 27: 1164–1173. 10.1177/0883073812448533 22764181 PMC3743553

[23] Cañizares J, Blanca JM, Navarro JA, Monrós E, Palau F, Moltó MD. dfh is a Drosophila homolog of the Friedreich's ataxia disease gene. Gene. 2000; 256: 35–42. 11054533 10.1016/s0378-1119(00)00343-7

[24] Kondapalli KC, Kok NM, Dancis A, Stemmler TL. Drosophila frataxin: an iron chaperone during cellular Fe-S cluster bioassembly. Biochemistry. 2008; 47: 6917–6927. 10.1021/bi800366d 18540637 PMC2664653

[25] Navarro JA, Llorens JV, Soriano S, Botella JA, Schneuwly S, Martínez-Sebastián MJ, et al Overexpression of human and fly frataxins in Drosophila provokes deleterious effects at biochemical, physiological and developmental levels. PLoS One. 2011; 6: e21017 10.1371/journal.pone.0021017 21779322 PMC3136927

[26] Anderson PR, Kirby K, Hilliker AJ, Phillips JP. RNAi-mediated suppression of the mitochondrial iron chaperone, frataxin, in Drosophila. Hum Mol Genet. 2005; 14: 3397–3405. 16203742 10.1093/hmg/ddi367

[27] Runko AP, Griswold AJ, Min KT. Overexpression of frataxin in the mitochondria increases resistance to oxidative stress and extends lifespan in Drosophila. FEBS Lett. 2008; 582: 715–719. 10.1016/j.febslet.2008.01.046 18258192

[28] Shidara Y, Hollenbeck PJ. Defects in mitochondrial axonal transport and membrane potential without increased reactive oxygen species production in a Drosophila model of Friedreich ataxia. J Neurosci. 2010; 30: 11369–11378. 10.1523/JNEUROSCI.0529-10.2010 20739558 PMC2943153

[29] Soriano S, Llorens JV, Blanco-Sobero L, Gutiérrez L, Calap-Quintana P, Morales MP, et al Deferiprone and idebenone rescue frataxin depletion phenotypes in a Drosophila model of Friedreich's ataxia. Gene. 2013; 521: 274–281. 10.1016/j.gene.2013.02.049 23542074

[30] Tricoire H, Palandri A, Bourdais A, Camadro JM, Monnier V. Methylene blue rescues heart defects in a Drosophila model of Friedreich's ataxia. Hum Mol Genet. 2014; 23: 968–979. 10.1093/hmg/ddt493 24105471

[31] Loewith R, Hall MN. Target of rapamycin (TOR) in nutrient signaling and growth control. Genetics. 2011; 189: 1177–1201. 10.1534/genetics.111.133363 22174183 PMC3241408

[32] Park J, Al-Ramahi I, Tan Q, Mollema N, Diaz-Garcia JR, Gallego-Flores T, et al RAS-MAPK-MSK1 pathway modulates ataxin 1 protein levels and toxicity in SCA1. Nature. 2013; 498: 325–331. 10.1038/nature12204 23719381 PMC4020154

[33] Rusten TE, Lindmo K, Juhász G, Sass M, Seglen PO, Brech A, et al Programmed autophagy in the Drosophila fat body is induced by ecdysone through regulation of the PI3K pathway. Dev Cell. 2004; 7: 179–192. 15296715 10.1016/j.devcel.2004.07.005

[34] Kawasaki H, Hirose S, Ueda H. A Simple and Quick Method to Isolate Nuclear Extracts from Pupae of Drosophila melanogaster. Cytotechnology. 2005; 49: 67–70. 10.1007/s10616-005-5414-3 19003064 PMC3449746

[35] Barcelo H, Stewart MJ. Altering Drosophila S6 kinase activity is consistent with a role for S6 kinase in growth. Genesis. 2002; 34: 83–85. 12324955 10.1002/gene.10132

[36] Healy DG, Falchi M, O’Sullivan SS, Bonifati V, Durr A, Bressman S, et al Phenotype, genotype, and worldwide genetic penetrance of LRRK2-associated Parkinson’s disease: a case-control study. Lancet Neurol. 2008; 7: 583–590. 10.1016/S1474-4422(08)70117-0 18539534 PMC2832754

[37] Imai Y, Gehrke S, Wang HQ, Takahashi R, Hasegawa K, Oota E, et al Phosphorylation of 4E-BP by LRRK2 affects the maintenance of dopaminergic neurons in Drosophila. EMBO J. 2008; 27: 2432–2443. 10.1038/emboj.2008.163 18701920 PMC2543051

[38] Katewa SD, Kapahi P. Role of TOR signaling in aging and related biological processes in Drosophila melanogaster. Exp Gerontol. 2011; 46: 382–390. 10.1016/j.exger.2010.11.036 21130151 PMC3058120

[39] Ravikumar B, Berger Z, Vacher C, O'Kane CJ, Rubinsztein DC. Rapamycin pre-treatment protects against apoptosis. Hum Mol Genet. 2006; 15: 1209–1216. 16497721 10.1093/hmg/ddl036

[40] Kapahi P, Chen D, Rogers AN, Katewa SD, Li PW, Thomas EL, et al With TOR, less is more: a key role for the conserved nutrient-sensing TOR pathway in aging. Cell Metab. 2010; 11: 453–465. 10.1016/j.cmet.2010.05.001 20519118 PMC2885591

[41] Layalle S, Arquier N, Léopold P. The TOR pathway couples nutrition and developmental timing in Drosophila. Dev Cell. 2008; 15: 568–577. 10.1016/j.devcel.2008.08.003 18854141

[42] Petiot A, Ogier-Denis E, Blommaart EF, Meijer AJ, Codogno P. Distinct classes of phosphatidylinositol 3’-kinases are involved in signaling pathways that control macroautophagy in HT-29 cells. J Biol Chem. 2000; 275: 992–998. 10625637 10.1074/jbc.275.2.992

[43] Kofman AE, McGraw MR, Payne CJ. Rapamycin increases oxidative stress response gene expression in adult stem cells. Aging. 2012; 4: 279–289. 22529334 10.18632/aging.100451 PMC3371763

[44] Robida-Stubbs S, Glover-Cutter K, Lamming DW, Mizunuma M, Narasimhan SD, Neumann-Haefelin E, et al TOR Signaling and Rapamycin Influence Longevity by Regulating SKN-1/Nrf and DAF-16/FoxO. Cell Metab. 2012; 15: 713–724. 10.1016/j.cmet.2012.04.007 22560223 PMC3348514

[45] Lodi R, Hart PE, Rajagopalan B, Taylor DJ, Crilley JG, Bradley JL, et al Antioxidant treatment improves in vivo cardiac and skeletal muscle bioenergetics in patients with Friedreich’s ataxia. Ann Neurol. 2001; 49: 590–596. 11357949

[46] Lynch DR, Lech G, Farmer JM, Balcer LJ, Bank W, Chance B, et al Near infrared muscle spectroscopy in patients with Friedreich's ataxia. Muscle Nerve. 2002; 25: 664–673. 11994959 10.1002/mus.10077

[47] Bonawitz ND, Chatenay-Lapointe M, Pan Y, Shadel GS. Reduced TOR Signaling Extends chronological Life Span via Increased Respiration and Upregulation of Mitochondrial Gene Expression. Cell Metab. 2007; 5: 265–277. 17403371 10.1016/j.cmet.2007.02.009 PMC3460550

[48] Zid BM, Rogers AN, Katewa SD, Vargas MA, Kolipinski MC, Lu TA, et al 4E-BP extends lifespan upon dietary restriction by enhancing mitochondrial activity in Drosophila. Cell. 2009; 139: 149–60. 10.1016/j.cell.2009.07.034 19804760 PMC2759400

[49] Wullschleger S, Loewith R, Hall MN. TOR signaling in growth and metabolism. Cell. 2006; 124: 471–484. 16469695 10.1016/j.cell.2006.01.016

[50] Bjedov I, Toivonen JM, Kerr F, Slack C, Jacobson J, Foley A, et al Mechanisms of life span extension by rapamycin in the fruit fly Drosophila melanogaster. Cell Metab. 2010; 11: 35–46. 10.1016/j.cmet.2009.11.010 20074526 PMC2824086

[51] Patel PH, Tamanoi F. Increased Rheb-TOR signaling enhances sensitivity of the whole organism to oxidative stress. J Cell Sci. 2006; 119: 4285–4292. 17038544 10.1242/jcs.03199

[52] Tettweiler G, Miron M, Jenkins M, Sonenberg N, Lasko PF. Starvation and oxidative stress resistance in Drosophila are mediated through the eIF4E-binding protein, d4E-BP. Genes Dev. 2005; 19: 1840–1843. 16055649 10.1101/gad.1311805 PMC1186182

[53] Auchère F, Santos R, Planamente S, Lesuisse E, Camadro JM. Glutathione dependent redox status of frataxin-deficient cells in a yeast model of Friedreich's ataxia. Hum Mol Genet. 2008; 17: 2790–2802. 10.1093/hmg/ddn178 18562474

[54] Pastore A, Tozzi G, Gaeta LM, Bertini E, Serafini V, Di Cesare S, et al Actin glutathionylation increases in fibroblasts of patients with Friedreich’s ataxia: a potential role in the pathogenesis of the disease. J Biol Chem. 2003; 278: 42588–42595. 12915401 10.1074/jbc.M301872200

[55] Tozzi G, Nuccetelli M, Lo Bello M, Bernardini S, Bellincampi L, Ballerini S, et al Antioxidant enzymes in blood of patients with Friedreich's ataxia. Arch Dis Child. 2002; 86: 376–379. 11970939 10.1136/adc.86.5.376 PMC1751091

[56] Marobbio CM, Pisano I, Porcelli V, Lasorsa FM, Palmieri L. Rapamycin reduces oxidative stress in frataxin-deficient yeast cells. Mitochondrion. 2012; 12: 156–161. 10.1016/j.mito.2011.07.001 21782979

[57] Sarkar S. Regulation of autophagy by mTOR-dependent and mTOR-independent pathways: autophagy dysfunction in neurodegenerative diseases and therapeutic application of autophagy enhancers. Biochem Soc Trans. 2013; 41: 1103–1130. 10.1042/BST20130134 24059496

[58] Turpaev KT. Keap1-Nrf2 signaling pathway: mechanisms of regulation and role in protection of cells against toxicity caused by xenobiotics and electrophiles. Biochemistry (Mosc). 2013; 78: 111–126. 23581983 10.1134/S0006297913020016

[59] Bryan HK, Olayanju A, Goldring CE, Park BK. The Nrf2 cell defence pathway: Keap1-dependent and-independent mechanisms of regulation. Biochem Pharmacol. 2013; 85: 705–717. 10.1016/j.bcp.2012.11.016 23219527

[60] Carracedo A, Pandolfi PP. The PTEN-PI3K pathway: of feedbacks and cross-talks. Oncogene. 2008; 27: 5527–5541. 10.1038/onc.2008.247 18794886

[61] O'Reilly KE, Rojo F, She QB, Solit D, Mills GB, Smith D, et al mTOR inhibition induces upstream receptor tyrosine kinase signaling and activates Akt. Cancer Res. 2006; 66: 1500–1508. 16452206 10.1158/0008-5472.CAN-05-2925 PMC3193604

[62] Simonsen A, Cumming RC, Brech A, Isakson P, Schubert DR, Finley KD. Promoting basal levels of autophagy in the nervous system enhances longevity and oxidant resistance in adult Drosophila. Autophagy. 2008; 4: 176–184. 18059160 10.4161/auto.5269

[63] Kaplan B, Qazi Y, Wellen JR. Strategies for the management of adverse events associated with mTOR inhibitors. Transplant Rev. 2014; 28: 126–133. 10.1016/j.trre.2014.03.002 24685370

[64] Kearney M, Orrell RW, Fahey M, Pandolfo M. Antioxidants and other pharmacological treatments for Friedreich ataxia. Cochrane Database Syst Rev. 2012; 4: CD007791 10.1002/14651858.CD007791.pub3 22513953

