# Supplementary figures and images for: TORC1 Inhibition by Rapamycin Promotes Antioxidant Defences in a Drosophila Model of Friedreich’s Ataxia
Source: PLoS One. 2015 Jul 9;10(7):e0132376. doi: 10.1371/journal.pone.0132376 (PMC4497667; doi:10.1371/journal.pone.0132376)

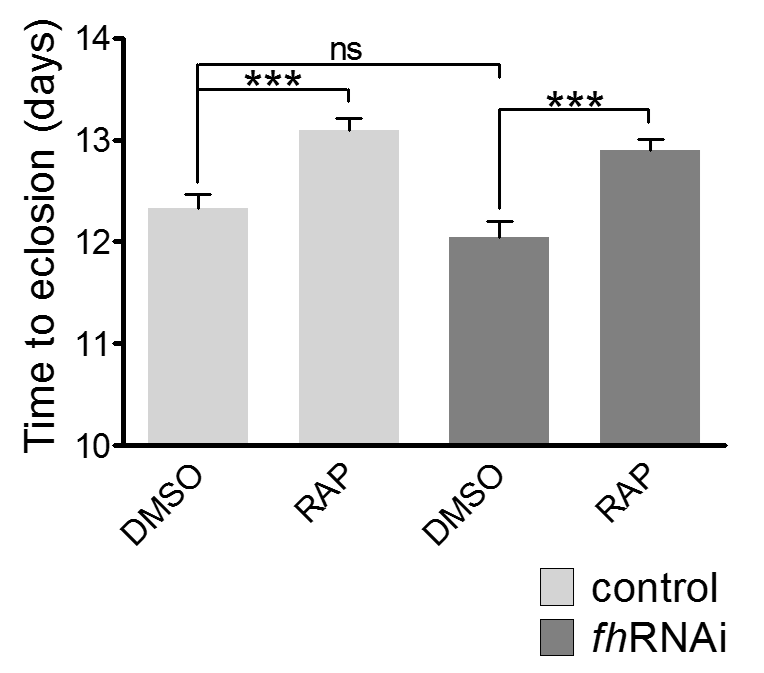

Supplement: S1 Fig — The time needed by individuals to eclose from the puparium was measured. The day the crosses were made was established as day zero. Parental flies were maintained in these vials for 2 days and then were removed. The results indicate the average time, in days, needed by individuals of F1 to complete the preadult development. Control (y1w*; actin-Gal4 flies), fhRNAi (UAS-fhRNAi; actin-Gal4 flies). ns: non-significant, ***P<0.001. (TIF) [file pone.0132376.s001.tif]

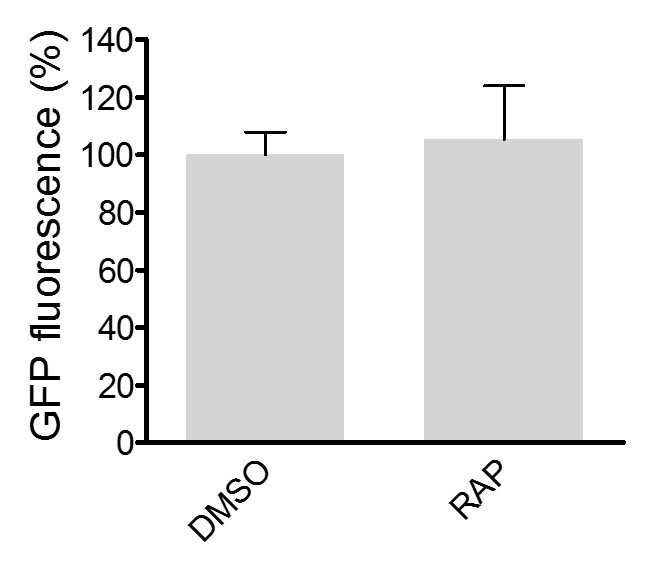

Supplement: S2 Fig — Fluorescence from thirty 7-day-old males expressing GFP in a ubiquitous pattern (UAS-GFP; actin-Gal4) was measured as previously described in [29]. (TIF) [file pone.0132376.s002.tif]

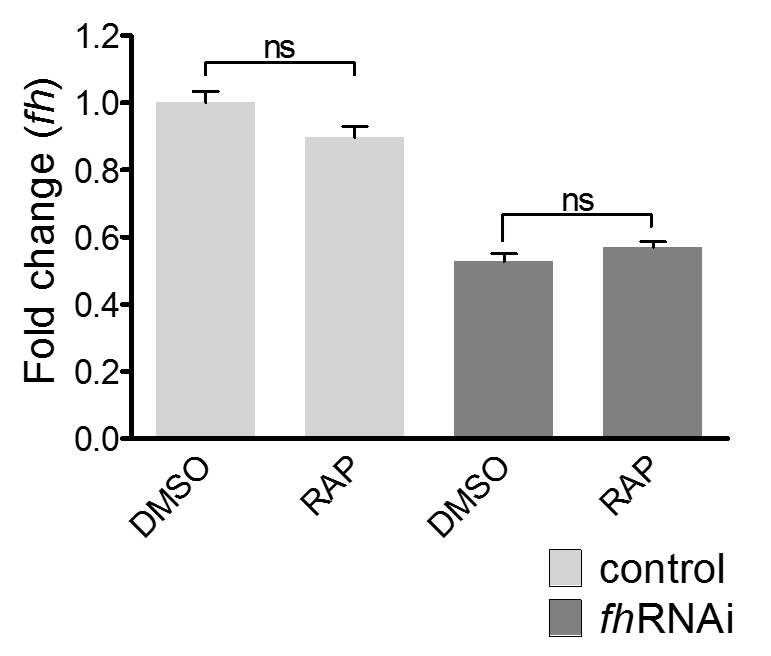

Supplement: S3 Fig — Control (y 1 w*; actin-Gal4 flies), fhRNAi(UAS-fhRNAi; actin-Gal4 flies). ns: non-significant. (TIF) [file pone.0132376.s003.tif]

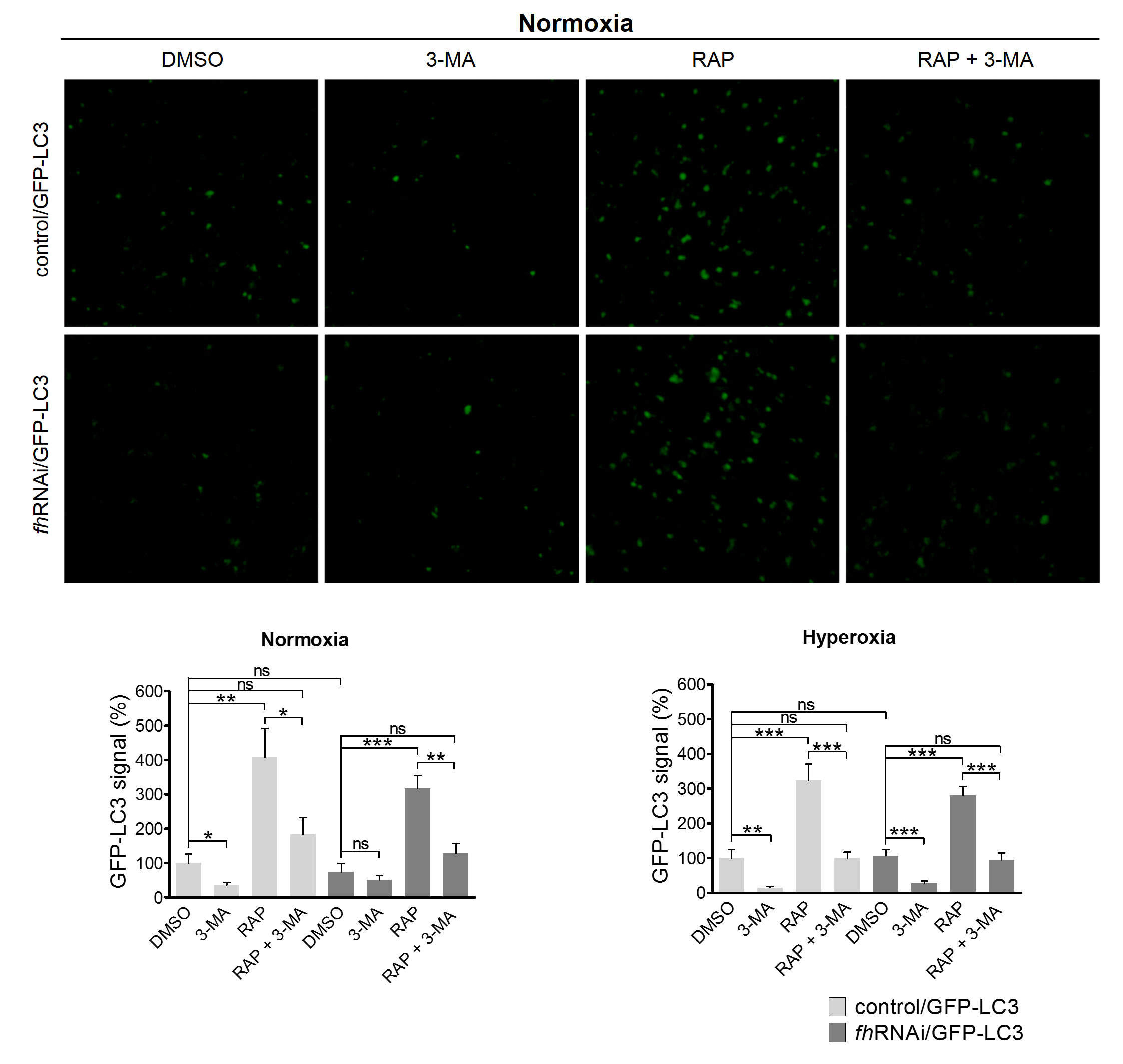

Supplement: S4 Fig — ns: non-significant, *P<0.05, **P<0.01, ***P<0.001. control/GFP-LC3 (UAS-GFP-LC3/+; Nos-Gal4/+) and fhRNAi/GFP-LC3: (UAS-GFP-LC3/UAS-fhRNAi; Nos-Gal4/+) (TIF) [file pone.0132376.s004.tif]

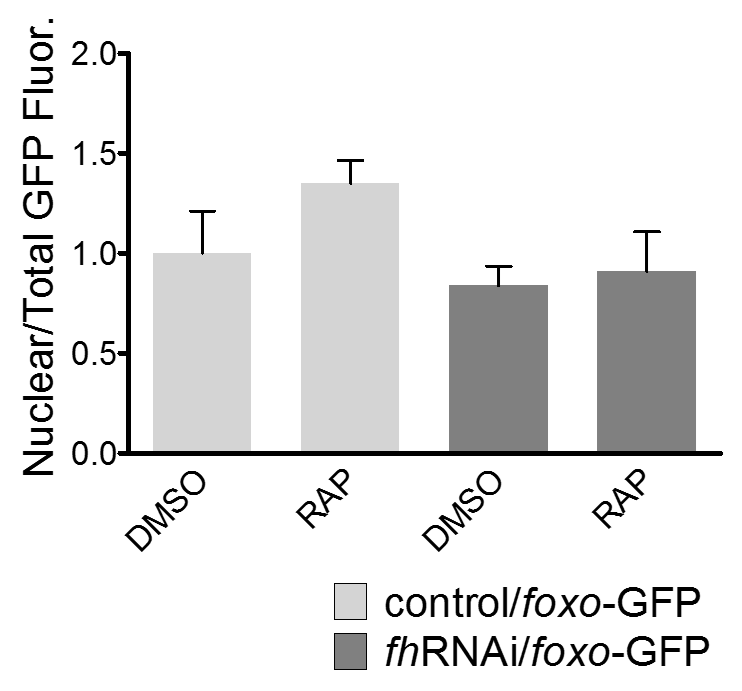

Supplement: S5 Fig — control/foxo-GFP (control flies expressing a foxo allele tagged with the GFP) and fhRNAi/foxo-EGFP: (fhRNAi flies expressing a foxo allele tagged with the GFP). (TIF) [file pone.0132376.s005.tif]

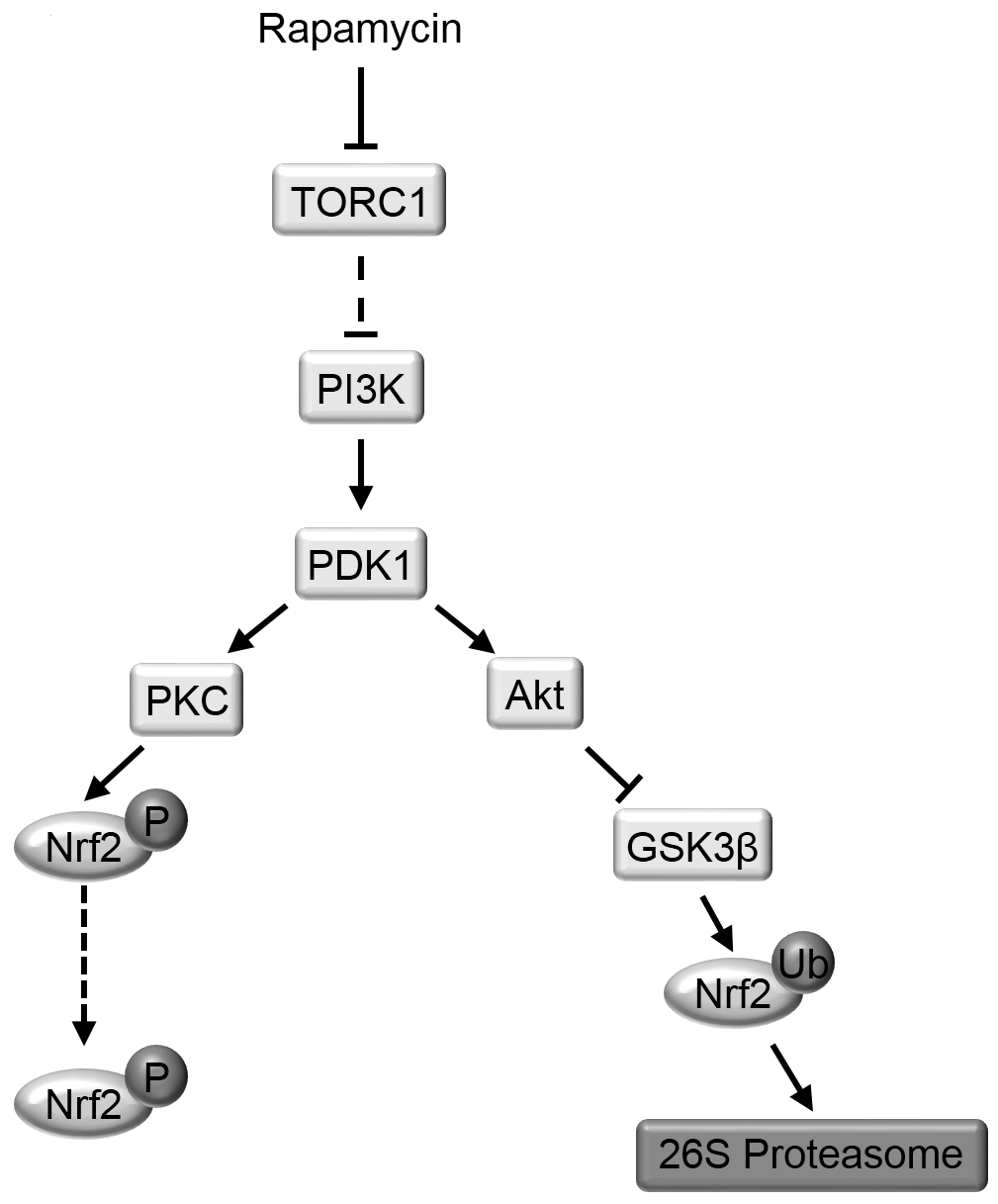

Supplement: S6 Fig — Rapamycin might increase Nrf2 activity by mechanisms depending on PKC and GSK3β, triggered by a TORC1 negative feedback loop which may increase PI3K-Akt signalling. (TIF) [file pone.0132376.s006.tif]

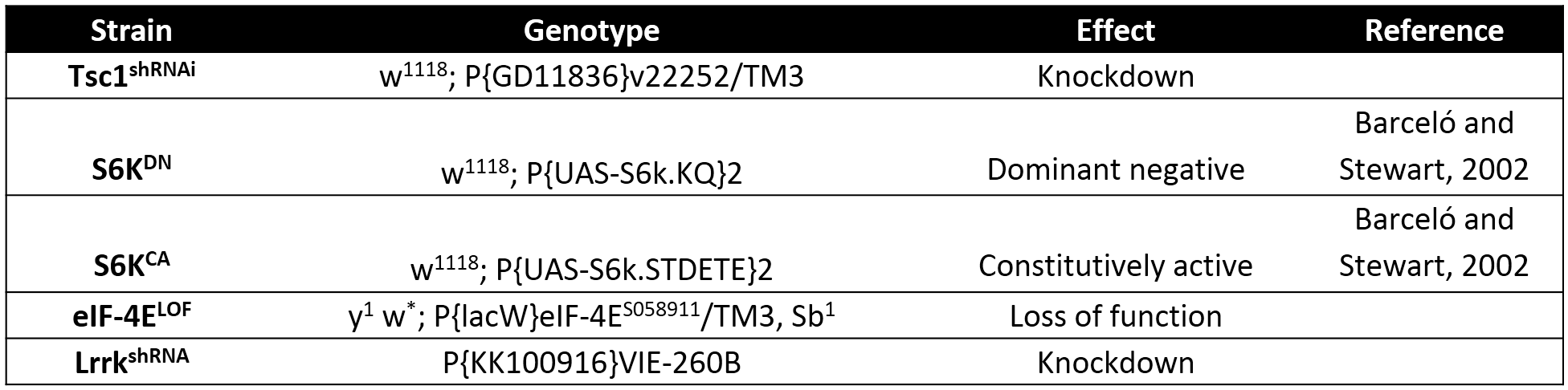

Supplement: S1 Table — (TIF) [file pone.0132376.s007.tif]
